# The experience of hate incidents across racial and ethnic groups during the COVID-19 pandemic

**DOI:** 10.3389/fpubh.2022.982029

**Published:** 2022-12-12

**Authors:** Carolyn A. Fan, KeliAnne K. Hara-Hubbard, Wendy E. Barrington, Barbara Baquero

**Affiliations:** ^1^Department of Health Systems and Population Health, University of Washington School of Public Health, Seattle, WA, United States; ^2^Health Promotion Research Center, Department of Health Systems and Population Health, University of Washington School of Public Health, Seattle, WA, United States; ^3^Center for Anti-Racism and Community Health, Department of Health Systems and Population Health, University of Washington School of Public Health, Seattle, WA, United States; ^4^Department of Child, Family, and Population Health Nursing, University of Washington School of Nursing, Seattle, WA, United States; ^5^Department of Epidemiology, University of Washington School of Public Health, Seattle, WA, United States

**Keywords:** racism, discrimination, hate incidents, hate crimes, race, violence, COVID-19

## Abstract

**Introduction:**

Racism is a root cause of ill health for communities of color, and hate incidents are one manifestation of racism. Marginalized racial and ethnic groups, including but not limited to Asian Americans, have been the target of highly publicized violence, hate, and discrimination which has been amplified during the COVID-19 pandemic.

**Objectives:**

This paper investigates (1) the prevalence of hate incidents across racial and ethnic groups, and (2) the relationship between race and ethnicity and hate incidents during the first year of the COVID-19 pandemic. We also seek to (3) situate study findings within theories of racism.

**Methods:**

This study utilizes national data from the Understanding America Study (UAS) COVID-19 Longitudinal Survey from June 10, 2020 to March 30, 2021 (*n* = 8,436). Hate incidents in six categories were examined: being treated with less courtesy, receiving poorer service, others acting as if they were not smart, others acting as if they were afraid of them, being threatened or harassed, and experiencing any of the previous categories of hate incidents. Main analyses were conducted *via* population averaged logistic panel regression.

**Results:**

The majority of members of all six marginalized racial and ethnic groups reported at least one hate incident during the first year of the COVID-19 pandemic. In addition, all marginalized racial or ethnic groups had statistically significant higher odds of experiencing at least two categories of hate incidents compared to white individuals. Asian, AI/AN, Black, and Multiracial groups had significantly higher odds of experiencing each category of hate incident. All marginalized racial and ethnic groups had significantly higher odds of receiving poorer service and others acting as if they were afraid of them.

**Conclusion:**

All marginalized racial and ethnic groups experienced significant levels of hate incidents within the first year of the COVID-19 pandemic. The public health community must continue to research, monitor, treat, and prevent hate incidents as a public health issue while recognizing the social and historical contexts of structural and interpersonal racism in the US.

## Introduction

Since the beginning of the COVID-19 pandemic in the United States (US), anti-Asian hate has been on the rise (1–3). A number of high-profile acts of violence, including the March 2021 shooting in Atlanta, GA, have deeply impacted Asian American communities. According to a 2021 Pew Research poll, 32% of Asian American adults report fears of being threatened or attacked, and 81% say that they believe that violence against them is increasing (4). From March 2020 to December 2021, nearly 11,000 hate incidents against Asian Americans and Pacific Islanders (AAPIs) have been reported to Stop AAPI Hate, a community and academic-based reporting site (3).

In this paper, we rely on the term “hate incident” because of its use in the national dialogue by community-based organizations (such as Stop AAPI Hate), news sites, and the general public during the COVID-19 era (5). As such, we hope this research will be more accessible to community organizations and members of the public who wish to prevent and address hate incidents. Unlike hate crimes, which are criminal and often violent acts motivated by bias, hate incidents represent a wider array of discriminatory acts (5, 6). Hate incidents do not have to meet legal definitions of a crime and therefore are not limited to violence, threats, or property damage (5, 6). In addition, the term allows for examination of hate outside the framework of the criminal and carceral systems. This is important because these systems place additional harm on marginalized communities, which some community leaders believe leads to police violence, higher incarceration rates, fear, and an investment in policing and incarceration instead of community resources (7–9). By relying solely on the criminal system to prevent and solve hate incidents, communities may be exposed to a double jeopardy of safety concerns–experiencing harm from both hate incidents and encounters with police attempting to address these incidents.

Hate incidents against Asian Americans have rapidly risen in the US, and this spike can be linked to the socio-political discourse around the COVID-19 pandemic, which began in early 2020. COVID-19 was deemed the “Chinese Virus” in the media on the federal level and beyond, thus blaming people of Asian descent for a global pandemic (10–13). This aligns with a long history of stigmatization of marginalized groups during major disease outbreaks, such as Africans and Ebola (14, 15), gay and bisexual men and HIV/AIDS (16, 17), and Asian Americans and SARS (15, 18). This stigmatization of Asian Americans directed the US public's fear, anger, and distrust around COVID-19 toward this group, fueling a surge of hate incidents and racist rhetoric. An analysis of US race-related Tweets comparing November 2019 (pre-COVID-19 pandemic) to March 2020 found that negative Tweets about Asians increased by 68.4%, making up approximately one out of six of total negative tweets in March 2020 (13). This national narrative has persisted, with one national poll finding that as of June 2021, most Americans (58%) believed that COVID-19 was designed in a Chinese laboratory (19). According to a national poll by the LAAUNCH Foundation, even more people blame individuals of Asian descent for the COVID-19 pandemic in 2022 than in 2021 (20).

Yet, these racist beliefs and attacks are not new. It is imperative to recognize that the underlying forces of structural and interpersonal racism existed before the COVID-19 pandemic. Racism is deeply ingrained in our systems, institutions, and interpersonal interactions (21). It is rooted in anti-Blackness, anti-Indigeneity, and the continued impacts of imperialism, settler colonialism, and slavery. For Asian Americans, structural and interpersonal racism and violence can be traced back for centuries (22, 23). Examples range from Yellow Peril and the Chinese Exclusion Act, Japanese Incarceration during WWII, the murder of Vincent Chin, post-9/11 hatred against Muslim Americans and South Asians, to the deportation of Southeast Asian refugees who originally migrated to the US due to American wars abroad (23).

Anti-Asian racism in the COVID-19 context is intertwined with other systems of oppression which facilitate this negative racialization. For instance, orientalism is the stereotyped way in which those in the “West” otherize, dehumanize, and take away power from the “East,” as informed by imperialism and colonialism (23–25). Orientalism can be clearly seen in the racialization of the COVID-19 virus (25). Xenophobia also interacts with racism; Asian Americans are viewed as “perpetual foreigners,” even though many Asians have been in the US for generations (10). This also aligns with racial triangulation theory, which posits that racial groups are not only deemed inferior or superior, but also as outsiders or insiders (10, 26, 27). Asian Americans, in particular, are seen as outsiders to US society (10, 26, 27). Finally, racial capitalism plays a role. Racial capitalism explains how racism and capitalism reinforce one another, as well as a recognition of how race is central to hierarchy in capitalist economies (28, 29). For instance, the aforementioned example of the murder of Vincent Chin was motivated by anti-Japanese and anti-Asian sentiment during a time when major layoffs in the US automotive industry were blamed on the success of Japanese companies, illustrating how socioeconomic and political factors are crucial parts of hate incidents (10, 30). Although socioeconomic factors are deeply interwoven into racial health disparities, the distinct experience of institutional, cultural, and interpersonal racism—outside of socioeconomic factors—is at the root of racial differences in health outcomes (31–34).

Even before the pandemic began in 2019, a vast majority of Asian American adults (76%) reported experiencing discrimination because of their race or ethnicity (35). However, Asian Americans are not the only ones who have experienced elevated hate during the pandemic. Black Americans have experienced years of highly publicized, racially motivated violence and murders committed by police (36, 37). The epidemic of missing and murdered Indigenous women, girls, and Two-spirit people has continued, as highlighted by the Urban Indian Health Institute and other researchers (38–40). A report from the Human Rights Campaign highlights that 2020 and 2021 were the deadliest years thus far for transgender and gender diverse people, especially for Black and Latinx transgender women (22). Over half of Native American, Black, and Hispanic workers have jobs which require them to work in close proximity to others during the pandemic, increasing their potential exposure to COVID-19 (41, 42). For example, there have been numerous reports of frontline workers such as healthcare, grocery store, and transportation workers attacked on the job due to COVID-19-related disputes (43). Theoretically, greater exposure to others could potentially increase exposure to hate incidents and violence during a time of heightened public fear, anger, and distrust.

These examples of discrimination and violence highlight how hate incidents can be considered both as social determinants of health (44) and as health outcomes themselves. Hate incidents can be health outcomes, as violent acts which result in bodily harm or death (44). Hate incidents can also impact the health of communities, with one act of hate potentially having a spillover health effects on members of a marginalized racial group as a whole (37). Exposure to hate incidents, as well as the anticipation of hate incidents, can act as social determinants, influencing health through pathways of psychosocial stress (44, 45). For instance, the weathering hypothesis theorizes that the stress of continued exposure to inequality results in health disparities (45). Hate incidents during the COVID-19 pandemic may pose even greater risks, with the potential for the spread of the disease during physical harassment due to close proximity, as well as posing a greater burden on already overwhelmed health systems. Although there is research highlighting the disproportionate mental health impact of hate incidents on Asian Americans during the COVID-19 pandemic (46, 47), it is important to measure the extent to which all marginalized racial and ethnic groups are impacted by hate incidents.

We use the term “marginalized racial and ethnic groups” in this paper to call out the act of marginalization enacted upon communities of color by systems dominated by white supremacy (48). This term and the concept of marginalization has been utilized in other papers on health inequities (33, 49, 50). Marginalized racial and ethnic groups include people of color of various backgrounds, who are subject to structural racism and discrimination. As such, all marginalized racial and ethnic groups are vulnerable to the deleterious effects of racism, and hate incidents occurring during the COVID-19 pandemic across all marginalized racial and ethnic groups are deserving of attention from the public health community. However, few other studies have investigated the experience of hate incidents during the COVID-19 pandemic across multiple racial and ethnic groups (51). Many studies focus only on COVID-related discrimination, while others may focus only on Asian Americans or a limited number of racial/ethnic groups (1, 51).

This study fills this gap by investigating the relationship between race and ethnicity and hate incidents in the year after the start of the COVID-19 pandemic in the US. Utilizing publicly available data from the University of Southern California's Understanding America Study's (UAS) COVID-19 Longitudinal Survey (47), we investigated three aims. First, we describe the prevalence and distribution of hate incidents across racial and ethnic groups during the start of the COVID-19 pandemic. Second, we evaluate our primary research aim: evaluate if members of six marginalized racial and ethnic groups [Asian, American Indian and Alaskan Native (AI/AN), Black, Hispanic, Multiracial, Native Hawaiian and Pacific Islander (NH/PI)] have significantly higher odds of experiencing six categories of hate incidents compared to white participants (being treated with less courtesy, receiving poorer service, others acting as if they were not smart, others acting as if they were afraid of them, being threatened or harassed, and experiencing any of the previous categories of hate incidents). We hypothesize that Asian Americans and all other marginalized racial and ethnic groups included in this study will have significantly higher odds of experiencing hate incidents during the COVID-19 pandemic compared to white participants. Finally, we seek to situate our study findings within theories of racism and white supremacy, as well as the sociopolitical context of the study time period.

## Methods

### Data source

The UAS COVID-19 Longitudinal Survey is part of the larger UAS. The UAS is a national, probability-based online panel of adults in the US which began in 2014 (52). Participants were recruited *via* address-based random sampling; with ~9,000 participants and 7,400 households participating (52). Any adults 18 years of age and older in a contacted household who can understand English or Spanish were eligible to participate. Participants respond to surveys online, and households without internet access were provided internet and a tablet or computer for the duration of the study (52). Participants are compensated $20 for every 30 min of survey time.

UAS began the COVID-19 longitudinal survey on March 10, 2020, when current UAS participants were asked to opt in to the COVID-19 survey (47). Survey waves occurred every 14- days, with participants having up to an additional 14 days to respond to the survey. Therefore, each survey represents experiences of a panel participant over the last 14–28-days since their previous survey (47). In this study, 18 waves in total are included, representing participants' experiences from June 10, 2020 to March 30, 2021. Waves 1–6 and 9 were excluded because they did not include the main outcome questions about hate incidents. Data for wave 26 and beyond were excluded to ensure consistency in the time frame asked by the survey questions, because the survey switched from biweekly to monthly and stand-alone surveys. This study includes *n* = 8,436 unique participants and *n* = 155,472 observations.

### Variables

#### Outcome: Hate incidents

The UAS' score survey modules, which are repeated in each survey wave, include questions about experiences of discrimination and hate (53). The survey questions below encompass five different types of hate incidents described below, including but not limited to being targeted by violence or harassment.

The survey asks participants, “*Since [date of previous survey], how often have any of the following things happened to you in your day-to-day life because of your actual or perceived race, ethnicity, age, gender, health, income, education, religion, or some other personal characteristic*?” The following situations are provided: (a) “*You were treated with less courtesy or respect than other people*,” (b) “*You received poorer service than other people at restaurants or stores*,” (c) “*People acted as if they thought you were not smart*,” (d) “*People acted as if they were afraid of you*,” and (e) “*You were threatened or harassed*.” Participants can respond with “*Almost every day*,” “*At least once a week*,” “*A few times a month*,” “*Once a month or less*,” or “*Never*.” These questions were adapted from the Everyday Discrimination Scale (Short Version) (EDS) (54), a widely used scale which is validated among adults of multiple race and ethnicities (55, 56). Hate incidents and everyday discrimination overlap in definition and can be similar. We interpreted the EDS as various categories of hate incidents, in order to align with national dialogue around hate incidents and hate crimes during the COVID-19 pandemic. Previously in the literature, scholars have categorized results from the EDS as hate-motivated crimes or incidents (2).

Participants were also asked to choose up to two main reasons for all the hate incidents they selected previously. We chose to include all hate incidents for analysis, even if participants attributed the incident to an identity other than their race or ethnicity. This was done to evaluate the distribution of all hate incidents across racial and ethnic groups. A key principle of Public Health Critical Race Praxis (PHCRP) is the primacy of racialization, what states that racialization is foundational to inequities in the US (57). Furthermore, following tenets of PHCRP, Intersectionality (58–60), and Critical Race Theory (61), we believe that racialization is inextricable from participants' experience of other social identities (e.g., gender, age, income). Therefore, even hate incidents that can be attributed to another identity are still shaped by how a person is racialized. Respondents to the EDS may struggle to attribute their experiences with discrimination to a single identity; one study found that 43% of participants had difficulty choosing one main reason for their discrimination when filling out the EDS (62). Therefore, when participants must choose one reason for the discrimination they experience, results may be underestimated or biased (62). To prevent these issues, scales have been developed that are “attribution-free,” instead basing analyses on the self-report of sociodemographic identities in a separate section of the survey, similar to how we approached our analysis (63).

For purposes of analysis, all hate incident outcome variables were dichotomized into “0 = No” for participants who responded “Never,” and “1 = Yes” for participants who reported any frequency of hate incidents since their last survey. An additional outcome variable was created which described if a participant experienced any of the above five categories of hate incidents, resulting in six total outcome variables. This variable was also dichotomized; if a participant selected “Never” for all five hate incident categories in the EDS, they were labeled as “0 = No.” Participants that selected a response other than “Never” for one or more hate incident categories were labeled as “1 = Yes.”

#### Exposure: Race/ethnicity

Demographic variables, including race and Hispanic ethnicity, are collected quarterly as part of the wider UAS study, and are included in the longitudinal dataset (53). In the UAS data, racial categories include white, Asian, Black, AI/AN, and NH/PI. Participants self-select their racial and ethnic group and are able to check multiple categories; those who did so are categorized as Multiracial. The UAS also asks for Hispanic ethnicity separately from race. In this analysis, participants were placed into seven exclusive racial and ethnic categories: non-Hispanic Asian, non-Hispanic AI/AN, non-Hispanic Black, non-Hispanic Multiracial, non-Hispanic NH/PI, non-Hispanic white, and Hispanic. In this paper, we will refer to these groups from this point forward as Asian, AI/AN, Black, Multiracial, NH/PI, white, and Hispanic, respectively. In this study, we conceptualize one's race and/or ethnicity as exposing them to racialization and multi-level forms of racism throughout their lives. Racialization, or the process in which people or groups are seen and defined in racial terms by others (64), patterns the way that a person experiences and is exposed to racism, discrimination, and violence.

#### Covariates

Additional confounders were adjusted for based on their theoretical links to the exposure (race and ethnicity) and the outcome (experience of hate incidents). There may be additional, unadjusted confounders which were not collected by the UAS. Adjusted confounders were: binary gender (male, female), age (continuous), immigration status (non-immigrant, first generation immigrant, second generation immigrant, third generation immigrant, unknown), education status (less than high school, high school graduate or GED, some college—no degree, Associate degree, Bachelor's degree, Graduate degree), current working status (yes, no), and household income (< $30,000; $30,000–$59,999; $60,000–$99,999; $100,000–$149,999; $150,000 or greater).

### Analysis

Statistical analyses were conducted using STATA 16 (65). In aim 1, descriptive statistics calculated both the overall frequency and between frequency of each hate incident by racial and ethnic group; these were tabulated taking into account the panel nature of the data. The primary aim, aim 2, was tested with a series of population-averaged logistic panel regressions with robust standard errors and exchangeable correlation, adjusted for observed confounders. The panel regression model accounted for clustering by participant id and wave, and panel robust standard errors accounted for heteroskedasticity. The population-averaged logistic regression was selected because it provides the interpretation of the odds of a given category of hate incident for the average member of a given marginalized racial/ethnic group in comparison to the average white participant (the reference group), adjusted for the observed confounders listed above.

## Results

Table 1 describes the characteristics of the study sample. Participants were 64.51% non-Hispanic white, 17.02% Hispanic, 8.00% Black, 5.12% Asian, 4.37% Multiracial, 0.93% AI/AN, and 0.31% NH/PI. The average age was 51.27 (SD = 16.06). 59.10% of participants were women. Additional descriptors can be found in Table 1.

**Table 1:** Descriptive demographic frequencies of survey sample.

**Variable**	**Category**	**N(%) or mean (SD)**
Race and ethnicity	Non-Hispanic (NH) Asian	431 (5.1%)
	NH American Indian/Alaskan Native (AI/AN)	78 (0.9%)
	NH Black	673 (8.0%)
	Hispanic	1,432 (17.0%)
	NH Multiracial	368 (4.4%)
	NH Native Hawaiian/Pacific Islander (NH/PI)	26 (0.3%)
	NH white	5,428 (64.5%)
Gender	Female	4,973 (59.1%)
	Male	8,414 (41.0%)
Age		51.27 (±16.1)
Immigrant status	Non-immigrant	4,528 (53.8%)
	First generation	962 (11.4%)
	Second generation	1,278 (15.2%)
	Third generation	1,482 (17.6%)
	Unknown	308 (3.7%)
Citizenship status	US citizen	8,121 (96.5%)
	Non-US citizen	293 (3.5%)
Education	Less than high school	42 (5.4%)
	High school graduate or GED	1,342 (16.8%)
	Some college-no degree	1,827 (22.8%)
	Associate degree	1,165 (14.5%)
	Bachelor's degree	1,967 (24.6%)
	Graduate degree	1,021 (12.7%)
Currently working	No	3,919 (46.6%)
	Yes	5,380 (64.0%)
Essential worker	Yes	4,049 (49.4%)
	No	2,468 (30.1%)
	Unsure	1,675 (20.5%)
Household income	Less than $30,000	2,384 (28.3%)
	$30,000 to $59,999	2,568 (30.5%)
	$60,000–$99,999	2,463 (29.3%)
	$100,000-$149,999	1,485 (17.7%)
	$150,000 or greater	1,150 (13.7%)

### Aim 1: Prevalence of hate incidents across racial and ethnic groups

Tables 2, 3 show descriptive statistics of our main outcome variables: six categories of hate incidents. Table 2 demonstrates the percentage of the time that a hate incident was reported over the course of the study time period, by hate incident category as well as racial and ethnic group. Table 3 describes the number of participants who reported a given hate incident at least once sometime during the duration of the panel, by hate incident category as well as racial and ethnic group. Both tables demonstrate that marginalized racial and ethnic groups generally report elevated levels of experiencing hate incidents compared to white participants on the event level as well as on the respondent level, across the timespan of the panel.

**Table 2:** Number of hate incidents reported in last 14–28 days (since the time of the previous survey), by hate incident category and race/ethnicity (total *n* = 155,472 observations).

	**Less courtesy**	**Poorer service**	**Not smart**	**Afraid of you**	**Threatened or harassed**	**Any hate incident**
Asian	1,152 (14.5%)	894 (11.2%)	920 (11.6%)	833 (10.5%)	683 (8.6%)	1,410 (17.7%)
AI/AN	198 (14.8%)	178 (13.3%)	179 (13.4%)	163 (12.2%)	122 (9.1%)	233 (17.5%)
Black	1,748 (14.6%)	1,423 (11.9%)	1,511 (12.7%)	1,252 (10.5%)	746 (6.3%)	2,214 (18.6%)
Hispanic	2,773 (11.7%)	2,226 (9.4%)	2,500 (10.6%)	1,910 (8.1%)	1,452 (6.2%)	3,565 (15.1%)
Multiracial	838 (13.0%)	538 (8.4%)	734 (11.4%)	538 (8.4%)	363 (5.7%)	1,105 (17.2%)
NH/PI	96 (21.9%)	88 (20.1%)	72 (16.4%)	77 (17.5%)	44 (10.0%)	111 (25.3%)
White	8,491 (8.2%)	5,197 (5.0%)	8,153 (7.9%)	5,142 (5.0%)	3,670 (3.5%)	12,430 (12.0%)
Total	15,296 (9.8%)	10,544 (6.8%)	14,096 (9.1%)	9,915 (6.4%)	7,080 (4.6%)	21,068 (13.6%)

**Table 3:** Aim 1: Number of participants who reported at least one hate incident during the duration of the study (June 10, 2020 to March 30, 2021), by hate incident category and race/ethnicity (*n* = 8,436 participants).

	**Less courtesy**	**Poorer service**	**Not smart**	**Afraid of you**	**Threatened or harassed**	**Any hate incident**
Asian	225 (52.2%)	173 (40.1%)	187 (43.4%)	176 (40.8%)	138 (32.0%)	260 (60.3%)
AI/AN	40 (51.3%)	37 (47.4%)	36 (46.2%)	35 (44.9%)	32 (41.0%)	46 (59.0%)
Black	366 (54.4%)	326 (48.4%)	332 (49.3%)	277 (41.2%)	213 (31.7%)	428 (63.6%)
Hispanic	659 (46.0%)	540 (37.7%)	601 (42.0%)	470 (32.8%)	394 (27.5%)	787 (55.0%)
Multiracial	184 (50.0%)	137 (37.2%)	167 (45.4%)	139 (37.8%)	114 (31.0%)	217 (59.0%)
NH/PI	16 (61.5%)	14 (53.9%)	13 (50.0%)	12 (46.2%)	10 (38.5%)	17 (65.4%)
White	1,965 (36.2%)	1,385 (25.5%)	1,856 (34.2%)	1,323 (24.4%)	1,115 (20.5%)	2,588 (47.7%)
Total	3,455 (41.0%)	2,612 (31.0%)	2,443 (29.0%)	2,443 (28.82)	2,016 (23.9%)	4,343 (51.5%)

For instance, out of all of the surveys waves conducted over the course of the study time period, Asian participants reported being threatened or harassed 8.6% of the time, compared to white participants who reported the same hate incident 3.5% of the time (Table 2). AI/AN participants reported being threatened or harassed 9.1% of the time, Black participants 6.3% of the time, Hispanic participants 6.2% of the time, Multiracial participants 5.7% of the time, and NH/PI participants 10.0% of the time (Table 2).

Notably, the majority of participants in all marginalized racial and ethnic groups experienced at least one type of hate incident during the time period of the study (Table 3). 60.3% of Asian participants, 59.0% of AI/AN participants, 63.6% of Black participants, 55.0% of Hispanic participants, and 59.0% of Multiracial participants, and 65.4% of NH/PI participants reported at least one hate incident during the study time period, compared to 47.7% of white participants (Table 3). Around a third or more of all marginalized racial and ethnic group members experienced threats or harassment during the panel time period (Table 3). 32.0% of Asian participants, 41.0% of AI/AN participants, 31.7% of Black participants, 27.5% of Hispanic participants, 31.0% of Multiracial participants, and 38.5% of NH/PI participants reported being threatened or harassed, compared to 20.5% of white participants.

### Aim 2: Odds of experiencing hate incidents by marginalized race and ethnic group

Table 4 contains the results of the study's primary research question: Do marginalized racial and ethnic groups have significantly higher odds of experiencing various hate incidents during the first year of the COVID-19 pandemic compared to white participants? All following results describe the odds of an average member of a given marginalized racial and ethnic group experiencing a hate incident since the last survey compared to the average white participant.

**Table 4:** Aim 2: Adjusted odds of marginalized racial or ethnic group participant experiencing given hate incident in last 14–28 days compared to non-Hispanic white participant.

		**Less courtesy**	**Poorer service**	**Not smart**	**Afraid of you**	**Threatened or harassed**	**Any**
Asian	OR (RSE)	1.6^***^(0.2)	2.1^***^(0.2)	1.3^**^(0.1)	1.9^***^(0.2)	1.9^***^(0.2)	1.4^***^(0.1)
	95% CI	**1.4–2.0**	**1.7–2.6**	**1.1–1.6**	**1.5–2.4**	**1.5–2.4**	**1.2–1.7**
AI/AN	OR (RSE)	1.8^**^(0.4)	2.5^***^(0.5)	1.6^*^(0.4)	2.7^***^(0.6)	2.3^***^(0.6)	1.5^*^(0.3)
	95% CI	**1.2–2.7**	**1.6–3.8**	**1.1–2.5**	**1.8–4.2**	**1.5–3.7**	**1.0–2.1**
Black	OR (RSE)	1.6^***^(0.1)	2.1^***^(0.2)	1.4^***^(0.1)	2.0^***^(0.2)	1.3^*^(0.1)	1.5^***^(0.1)
	95% CI	**1.4–1.9**	**1.8–2.5**	**1.2–1.6**	**1.7–2.4**	**1.1–1.6**	**1.3–1.7**
Hispanic	OR (RSE)	1.1 (0.1)	1.4^***^(0.1)	1.0 (0.1)	1.3^**^(0.1)	1.0 (0.1)	1.1 (0.1)
	95% CI	1.0–1.3	**1.2–1.7**	0.9–1.2	**1.1–1.5**	0.8–1.2	0.9–1.2
Multiracial	OR (RSE)	1.6^**^(0.2)	1.6^***^(0.2)	1.4^***^(0.2)	1.8^***^(0.2)	1.5^*^(0.2)	1.5^***^(0.1)
	95% CI	**1.3–2.0**	**1.3–2.1**	**1.2–1.8**	**1.4–2.2**	**1.1–2.1**	**1.2–1.7**
NH/PI	OR (RSE)	2.1 (0.8)	3.8^***^(1.2)	1.7 (0.6)	3.6^***^(1.3)	2.4^*^(1.0)	1.6 (0.6)
	95% CI	1.0–4.6	**2.0–7.1**	0.8–3.5	**1.7–7.4**	**1.1–5.3**	0.8–3.5

Bolded: statistically significant odds (p-value 0.05);

* p-value 0.05,

** p-value 0.01,

*** p-value 0.001; OR, Odds Ratio; RSE, robust standard errors; CI, Confidence interval.

All racial and ethnic groups demonstrated significantly higher odds of experiencing hate incidents in multiple analyzed categories. A number of racial groups (Asian, AI/AN, Black, and Multiracial) demonstrated significantly higher odds in all six categories.

Namely, Asian participants had about twice the odds of receiving poorer service (OR = 2.07; 95% CI: 1.68–2.55; *p* ≤ 0.001), others acting afraid of them (OR = 1.90; 95% CI: 1.54–2.36; *p* ≤ 0.001), and being threatened or harassed (OR = 1.87; 95% CI: 1.48–2.37; *p* ≤ 0.001), as well as elevated odds of experiencing any hate incident (OR = 1.41; 95% CI: 1.19–1.67; *p* ≤ 0.001), and being treated as not as smart as others (OR = 1.31; 95% CI: 1.07–1.60; *p* ≤ 0.01).

AI/AN participants had nearly three times the odds of others acting afraid of them (OR = 2.72; 95% CI: 1.75–4.23; *p* ≤ 0.001), and more than twice the odds of receiving poorer service (OR = 2.48; 95% CI: 1.63–3.77; *p* ≤ 0.001) and being threatened or harassed (OR = 2.30; 95% CI: 1.45–3.66; *p* ≤ 0.001). They also had significantly elevated odds of being treated with less courtesy (OR = 1.79; 95% CI: 1.20–2.66; *p* ≤ 0.01), being treated as not as smart as others (OR = 1.64; 95% CI: 1.07–2.51; *p* ≤ 0.05), and experiencing any hate incident (OR = 1.45; 95% CI: 1.004–2.09; *p* ≤ 0.05).

Black participants had at least twice the odds of receiving poorer service (OR = 2.11; 95% CI: 1.80–2.48; *p* ≤ 0.001) and others acting afraid of them (OR = 2.00; 95% CI: 1.69–2.38; *p* ≤ 0.001). They also had significantly higher odds of being treated with less courtesy (OR = 1.62; 95% CI: 1.39–1.87; *p* ≤ 0.001), experiencing any hate incident (OR = 1.45; 95% CI: 1.27–1.65; *p* ≤ 0.001), being treated as not as smart as others (OR = 1.36; 95% CI: 1.16–1.58; *p* ≤ 0.001), and being threatened or harassed (OR = 1.30; 95% CI: 1.06–1.60; *p* ≤ 0.05).

Multiracial participants had significantly higher odds of others acting as if they were afraid of them (OR = 1.76; 95% CI: 1.41–2.19; *p* ≤ 0.001), receiving poorer service (OR = 1.64; 95% CI: 1.31–2.05; *p* ≤ 0.001), being treated with less courtesy (OR = 1.58; 95% CI: 1.30–1.91; *p* ≤ 0.01), being threatened or harassed (OR = 1.49; 95% CI: 1.09–2.05; *p* ≤ 0.05), experiencing any hate incident (OR = 1.46; 95% CI: 1.23–1.73; *p* ≤ 0.001), and being treated as not as smart as others (OR = 1.44; 95% CI: 1.19–1.76; *p* ≤ 0.001).

NH/PI participants demonstrated significantly higher odds in three hate incident categories. They had over three times the odds of receiving poorer service (OR = 3.76; 95% CI: 1.98–7.13; *p* ≤ 0.001) and others acting as if they were afraid of them (OR = 3.57; 95% CI: 1.72–7.39; *p* ≤ 0.001), as well as over two times the odds of being threatened or harassed (OR = 2.40; 95% CI: 1.08–5.32; *p* ≤ 0.05). Hispanic participants had significantly higher odds in two categories: receiving poorer service (OR = 1.40; 95% CI: 1.18–1.67; *p* ≤ 0.001) and others acting as if they were afraid of them (OR = 1.28; 95% CI: 1.10–1.49; *p* ≤ 0.01).

All marginalized racial and ethnic groups had significantly higher odds of receiving poorer service and others acting as if they were afraid of them. Five out of six of these groups had significant odds of being threatened or harassed (Asian, AI/AN, Black, Multiracial, and NH/PI). Four out of six of these groups (Asian, AI/AN, Black, and Multiracial) had significantly higher odds of experiencing the remaining hate incident categories: being treated with less courtesy and respect, others acting as if they were not smart, and experiencing any of the categories of hate incidents.

## Discussion

### Aims 1 and 2 discussion

This study demonstrates that during the first year of the COVID-19 pandemic, hate incidents were a significant and acute issue for individuals from marginalized racial and ethnic groups in comparison to white individuals. The prevalence of hate incidents was high, with the majority of members from marginalized racial and ethnic groups experiencing at least one type of hate incident during the study time period. Notably, Asian, AI/AN, Black, and Multiracial groups had significantly higher odds of experiencing every type of hate incidents examined in this study, but all marginalized racial and ethnic groups had significantly elevated odds in at least two hate incident categories. In addition, all six marginalized racial and ethnic groups had significantly higher odds of receiving poorer service and others acting as if they were afraid of them. Being threatened or harassed was also a crucial issue, with five out of six marginalized racial and ethnic groups experiencing significantly higher odds. Being threatened or harassed is the closest measured hate incident category to violence, and it has perhaps the most direct impact on physical, mental, and emotional health.

However, all hate incident categories have implications for the health of individuals and communities by making individuals feel unsafe or unwelcome in their day to day lives. The consequences of interpersonal hate incidents affect health through demonstrated theories such as weathering (45), embodied inequality (66), the minority stress model (67, 68), historical trauma (69), and the social-ecological model (70). Interpersonal hate incidents can cause poor health through psychological, biological, behavioral, and healthcare access pathways on the individual and collective level (32). These health issues include worse outcomes in depression, anxiety, PTSD, blood pressure, inflammation, allostatic load, and sleep (34).

### Aim 3: Theoretical and sociopolitical context of results

Overall, the results of this study reflect the reality of interpersonal hate, racism, and discrimination for marginalized racial and ethnic groups in the US during the COVID-19 pandemic. One might theorize that hate incidents during a pandemic would be minimal, since there are fewer opportunities to interact with others; however, this does not appear to be the case. Although Asian Americans have been in the spotlight for increased hate during the COVID-19 pandemic, particularly because many hate incidents against them are directly tied to COVID-19 itself, all marginalized racial and ethnic groups experienced hate incidents at elevated levels. Asian Americans had among the highest odds of being threatened or harassed, which could reflect these COVID-19-motivated hate incidents. Although hate incidents against other racial and ethnic groups may not have COVID-19 as a direct motivation, the COVID-19 pandemic nonetheless served as a backdrop for the hate incidents reported in this study's timeframe. In addition, the global recession, stay at home and social distancing orders, overwhelmed hospitals, and vaccine rollouts all likely shaped one's exposure to others, which subsequently shaped exposure to interpersonal hate incidents during the pandemic.

During the same time period, the US also underwent major sociopolitical shifts. Multiple events and trends relevant to racism and racial equity took place, including the Black Lives Matter protests, the continued murders of Black Americans at the hands of police, the 2020 Presidential election, the Capitol riots, the mass shooting in Atlanta, GA, and the Stop Asian Hate movement. These events likely shaped the way participants from marginalized racial and ethnic groups were perceived and racialized, as well as the nature and frequency of the hate directed toward them. In addition, racialized groups may have also been more vigilant toward acts of discrimination due to current events; this could have encouraged greater reporting of hate incidents and more groups speaking out about the racism they face in their day to day lives.

In addition, marginalized racial and ethnic groups across the country have come together through some of the largest organized protests against police violence, the carceral system, anti-Asian hate, immigrant detention centers, and more (71, 72). This community organizing presents a resistance to racist systems and institutions (73). Together, activists, community members, and other stakeholders seek to re-imagine our collective futures through anti-racist praxis and community organizing (73). This presents a threat to traditional norms of white supremacy. As theorized by Dr. Tema Okun, fear is one characteristic of white supremacy culture (74). White supremacy seeks to make marginalized racial and ethnic groups afraid (74). This is accomplished through interpersonal racism such as microaggressions, violence, and hate incidents (74). This can be extended further to other abuses of power, such as structural violence and carceral systems (74). As such, punishment is used to create fear, on internalized, interpersonal, and structural levels. In times such as COVID-19 and other major epidemics and pandemics, fear is especially heightened, which leads to discrimination and prejudice against stigmatized groups (15). For instance, Asian Americans have once again entered the realm of “Yellow Peril,” in which they are negatively racialized as dangerous to the health of the nation, outside of their perceived status as the “Model Minority” (10, 24). We theorize that this results in the depth and breadth of hate incidents experienced across marginalized racial and ethnic groups during the first year of the pandemic that we see in the outcomes of this study.

Although the common time period of the COVID-19 pandemic underlies the data in this study, we must also acknowledge that hate incidents are situated in different historical and social contexts for each distinct marginalized racial and ethnic group. In fact, the contexts are likely different for ethnicities within racial groups as well. Centuries of structural and interpersonal racism create iterative cycles of false stereotypes and biases, which otherize and de-humanize members of marginalized racial and ethnic groups. These cycles and contexts are all unique for different racial and ethnic groups, even if they result in the same broader hate incident. Take, for instance, the hate incident category “people acted as if they were afraid of you,” which was significant across all marginalized groups. For Asian Americans, this can be traced not only to COVID-19-based fears, but also to a long history of Asians being seen as dangerous and perpetually foreign, such as through “Yellow Peril” and post-9/11 stereotypes. This belies Asian Americans' perceived status as the “Model Minority” (22, 75). In fact, Asian Americans also had significantly higher odds of being seen as “not smart” compared to white Americans. These results reiterate the “Model Minority” label as a tool for white supremacy. It can be used to drive a wedge between Asian Americans and other people of color; however, it can also be quickly revoked once Asians pose a threat. For AI/ANs, Black Americans, Hispanics, Multiracial Americans, and NH/PIs, different stereotypes and biases underpin the same hate incident. For instance—AI/AN stereotypes of the “savage Indian” are rooted in colonialization (76). Stereotypes of Black Americans as violent and dangerous are born from the institution of slavery (77). Hispanic and Latin Americans were labeled as bringing drugs, crime, and rape during the 2016 presidential election due to racism and xenophobia (78). In addition, intersecting power structures (e.g., cisheterosexism, capitalism, ablism) may change the experience of these hate incidents if one has multiple marginalized identities. Ultimately, although these contexts and experiences vary across racial, ethnic, and cultural backgrounds, the deep roots of white supremacy and racism nonetheless underpin all of these acts of hate.

### Limitations and strengths

There are a number of limitations to this study. As with any non-randomized study, unmeasured confounders may introduce bias. Both the NH/PI and AI/AN groups had small sample sizes, creating wide confidence intervals. These confidence intervals at times overlapped with the confidence intervals of the other groups. In addition, the UAS survey is conducted in English and Spanish only. Therefore, those who speak languages other than English and Spanish were excluded from the study. As a result, the odds ratio estimations could be conservative, as language is one way in which groups are racialized and could be a crucial motivator for hate. In particular, many hate incidents against Asian Americans have targeted elderly, low-income, immigrant individuals who primarily speak languages other than English.

We examined six marginalized racial and ethnic groups; however, we did not disaggregate these groups further by ethnicity. For instance, the disaggregation of the Asian racial group may result in varying outcomes for East Asian vs. South Asian and Southeast Asian ethnicities because of the different ways these groups are racialized. Even though COVID-19 is linked directly to sinophobic rhetoric, other Asian ethnic groups have been targeted for violence and hate as well.

It is also possible that misclassification bias could have occurred. Standard racial questions are often ill-equipped to capture the full range of racialization in the US. For example, individuals from the Middle East and North Africa are often told to check “white” as a race despite being marginalized in the US. The Asian racial group is also widely diverse, but often misinterpreted as representing only East Asians. This could result in non-East Asians selecting other racial categories. In addition, Hispanic ethnicity is asked separately from race. In this analysis, Hispanics of any racial background were put into one category, potentially impacting that group's outcomes. Likewise, the Multiracial group is extremely diverse, representing people of various racial and ethnic backgrounds. As a result, it is difficult to know exactly how participants in this group have been racialized.

Another limitation is our analytic decision to utilize the EDS in an atypical way. The EDS asks respondents to choose a main reason for their experiences with discrimination from a list of social identities or other physical characteristics. However, based on precedent from other surveys (62), we chose to include all hate incidents for analysis, even if an identity not related to race or ethnicity was selected for attribution. Our aim for this study was to evaluate the distribution of hate incidents across different racial and ethnic groups, no matter the perceived cause. In addition, our reasoning includes the primacy of race and the inextricability of race from other social identities, based on theories such as intersectionality (60) and PHCRP (57). However, others have argued that respondents from marginalized racial and ethnic groups may not always perceive their race or ethnicity as a reason for hate incidents, even when they are allowed to select multiple reasons for their mistreatment (62).

The UAS may also be impacted by biases typical to self-administered, large survey panels, such as non-response bias. However, its nationwide, random sampling recruitment process which emphasized inclusion (e.g., providing internet access) likely reduced this. The UAS reports a recruitment rate of 13–15%, a rate similar to or higher than other similar online panels (47). The UAS has also found that its data quality is similar to traditional national surveys (47). Social desirability bias may still be present, potentially resulting in the under-reporting of hate incidents. However, as noted in the discussion, the recent waves of awareness around hate incidents during the COVID-19 pandemic may have also encouraged more individuals to accurately report their experiences with interpersonal racism.

Finally, we cannot compare our estimates to a pre-COVID-19 pandemic era, as the main outcomes were not collected during the regular UAS. Therefore, we cannot conclude that these results are elevated or different from pre-COVID-19 times, but we can consider the results within the context of the pandemic.

There are a number of strengths of this study as well. This study leverages the UAS, a large, publicly available, national panel dataset. The sample is recruited at random through address-based sampling. In addition, the UAS intentionally recruits underrepresented groups, allowing for analysis of typically smaller groups. As a result, a total of six marginalized racial and ethnic groups were examined, including AI/AN, Multiracial, and NH/PI groups which are often considered as an “Other” racial category or lumped with another group. In particular, NH/PIs are often combined with the Asian racial group, potentially obscuring distinct results. Despite small sample sizes for some groups, statistically significant associations were still found, indicating strong associations. As a whole, a major strength of this study is the examination of multiple racial and ethnic groups, rather than just one singular racial group. This allows us to understand a broader picture of hate incidents during the COVID-19 pandemic. To our knowledge, it is the only research study examining broad experiences of hate across multiple marginalized racial and ethnic groups in the year following the start of the COVID-19 pandemic in the US.

### Future research

There are many opportunities for future research on the topic of hate incidents across racial and ethnic groups. Racial groups can be further disaggregated by ethnicity, as there may be differing experiences across ethnic groups. In addition, studies focusing on hate against AI/AN and NH/PI populations should be prioritized, as study results indicate high odds ratios for these two groups. Larger sample sizes could allow further analysis of these groups.

Other avenues of research include exploration of hate incidents as modified by intersecting identities. For instance, gender, immigration status, and age all may interact with race and ethnicity in unique ways because of the ways systems of oppression (i.e., sexism, xenophobia, and agism) interlock with and reinforce racism. Reports from Stop AAPI Hate indicate that hate incidents are 2.3 times more likely to be reported by women, and non-binary individuals are more likely to report various hate incidents such as shunning, being coughed or spat on, and receiving online harassment (3). As such, discrimination against women of color, as well as transgender and gender diverse people of color, are important areas of focus.

Future research should also examine the structural determinants of interpersonal hate incidents. In addition to examining the historical and social contexts of race and ethnicity-based hate and discrimination in greater detail, there are various structures and systems which contribute to interpersonal hate and violence. These structural factors may be considered forms of violence themselves (23), as well as contributing to acute forms of interpersonal violence. For instance, socioeconomic inequities and employment policies may contribute to marginalized racial and ethnic groups working in frontline jobs during the pandemic, exposing them not only to discrimination and violence but also to COVID-19 itself.

Finally, research should also examine potential protective factors against the ill effects of hate incidents, such as resilience, mutual aid, activism, and community care. Although people of color are systematically marginalized, there is also strength and power to be found within and across communities. This power can be galvanized to prevent and address hate incidents and racism.

## Conclusion

This study assessed the prevalence of hate incidents and the odds of experiencing a hate incident across all marginalized racial and ethnic groups during the COVID-19 pandemic. As demonstrated in this study, hate incidents have impacted all marginalized racial and ethnic groups in the first year of the COVID-19 pandemic. All marginalized racial or ethnic groups had statistically significant higher odds of experiencing at least two categories of hate incidents compared to white individuals: receiving poorer service and others acting as if they were afraid of them. Asian, AI/AN, Black, and Multiracial groups had significantly higher odds of experiencing each category of hate incident. While marginalized racial and ethnic groups all experienced hate incidents, the racialization and historical context leading to these incidents differs greatly.

These hate incidents have resounding effects, not only on the individuals targeted, but also on their communities and beyond. As the COVID-19 pandemic continues, hate incidents continue as well. In 2022, numerous acts of violence driven by racism have harmed communities of color across the country. These events, alongside our study's results, indicate a continued pattern of increased violence against marginalized racial and ethnic groups that must be addressed by public health professionals. It is of the utmost imperative that the public health community understands the nuanced social and historical roots of these hate incidents. The public health field can take action to disrupt hate incidents through continued research, monitoring, treatment, and prevention of hate incidents and their negative impacts on health.

## Data availability statement

The project described in this paper relies on data from survey(s) administered by the Understanding America Study, which is maintained by the Center for Economic and Social Research (CESR) at the University of Southern California. Publicly available datasets were analyzed and can be found here: https://uasdata.usc.edu/index.php.

## Author contributions

CF and BB collaborated on the conception and design of the study. CF extracted the data, conducted statistical analysis, and wrote the first draft of the manuscript. KH-H contributed to the introduction and conclusion. KH-H, WB, and BB provided feedback on the statistical analysis. CF, KH-H, WB, and BB contributed to the interpretation of analytical results, as well as multiple iterations of critical revision, and editing of the manuscript as a whole. All authors provide approval of publication and agree to be held accountable for all aspects of the manuscript.
